# Supplementary material for: Service user perspectives and experiences of their diagnosis of psychotic major depression: A qualitative study
Source: PLoS One. 2025 May 2;20(5):e0322868. doi: 10.1371/journal.pone.0322868 (PMC12047751; doi:10.1371/journal.pone.0322868)
Supplement: Attachment — Submitted filename: Comments of the reviewer .docx [file pone.0322868.s001.docx]

Service user perspectives and experiences of their diagnosis of psychotic major depression: a qualitative study

1. The topic is interesting, but
2. Why u choice this topic
3. In the introduction part, you should only focused about psychotic major depression
4. What tools do you used to assess psychotic major depression?
5. What is the difference between major depression disorder with psychotic features, schizoaffective disorder and psychotic major depression?
6. Is schizoaffective disorder or not?
7. To Dx a patient with psychotic major depression is based- on DSM-5 or not, if not by what criteria will be diagnosis with psychotic major depression?
8. Is new finding on the management of major depression disorder with psychotic features, psychotic major depression and schizoaffective disorder? If yes what is new finding on the management of psychotic major depression?
9. In conclusions part: Psychotic major depression should be actively considered as a differential diagnosis by healthcare professionals when assessing an individual for unipolar depression, it is not understand, to me my 1^st^ DDX will be schizoaffective disorder or bipolar 1 disorder with psychotic feature.
10. References, there problems on references examples Hales [11] suggests that because patients recognise that their thought patterns are unusual, they may attempt to conceal their symptoms from others…..
11. Which tools were used to assess barriers, and stigma…
12. Write your Source of population,Study population, and study unit
13. What sampling methods and technique were used to asses
14. Why only 10 people participated in the interviews?
15. Service user frustration with diagnostic instability and lack of certainty, what does it mean
16. In the Discussion part, there is no references, so put references and discuss with different studies
17. How they know whether the diagnoses were psychotic major depression or not?
18. What does it mean medical malpractice?
19. Why you exclude individuals above 64 years old from the study?
