# Supplementary material for: Service user perspectives and experiences of their diagnosis of psychotic major depression: A qualitative study
Source: PLoS One. 2025 May 2;20(5):e0322868. doi: 10.1371/journal.pone.0322868 (PMC12047751; doi:10.1371/journal.pone.0322868)
Supplement: Attachment — Submitted filename: Response to reviewers.docx [file pone.0322868.s002.docx]

**Response to Reviewers**

Many thanks to the editor for considering this paper, and thanks to the reviewer for their comments. Please see our responses below.

**Responses to editor comments**

**Editor comment:** Respond comprehensively to all reviewer comments, ensuring clarity on recruitment methods, diagnostic criteria, study population, and sampling strategies.

**Author response:** Addressed below.

**Editor comment:** Adhere to the COREQ checklist (or an equivalent) and upload the completed checklist within your response. This should ensure thorough reporting of objectives, sampling strategies, data collection, analysis, and discussions of bias and limitations.

**Author response:** Completed, with some details added to the manuscript.

**Editor comment:** Clarify conflicting points raised by the reviewers, particularly around diagnostic definitions, participant recruitment, and how the diagnosis was ultimately made.

**Author response:** Addressed below.

**Responses to reviewer comments**

**Reviewer comment:** The subjects were recruited who had formal recorded clinical diagnosis of psychotic major depression in the past three years. It appears the 38 former patients (“service users”) were identified and 10 agreed to participate. The study implies that PMD had been “diagnosed late” but we know nothing about that; we're all 38 former patients diagnosed late, or just the 10 who had been interviewed.

**Author response:** Apologies for this misunderstanding. We recruited 10 people with a diagnosis of PMD to explore their experiences of the diagnosis process. They did not have to be “diagnosed late” to be included, we included all people with a PMD diagnosis who agreed to participate. However, where patients reported a long delay between initial symptoms and PMD diagnosis, we explored this in depth to try to determine why this was. There is no definition of “late diagnosis” so we did not determine who this was applicable to, only explored the process for each person and tried to describe this. We have clarified this in the abstract and introduction.

**Reviewer comment:** Were there clinical differences between the 28 non participants and the 10 who agreed to participate?

**Author response:** As described, potential participants were recruited via a consent for contact registry and clinical referrals. Beyond PMD diagnosis and contact details, we did not have any clinical data on the potential participants and therefore cannot describe the clinical differences between those who did and did not agree to participate. We have added this as a limitation to the discussion.

**Reviewer comment:** At what point did the true diagnosis of PMD become apparent and what contributed to the appropriate diagnosis?

**Author response:** As described in the methods, participants needed to have a formal diagnosis of PMD according to their medical records. Beyond this, we have no knowledge of the clinical diagnosis history or process. This study focussed on the experience from a patient perspective.

**Reviewer comment:** The authors do a sterling job of representing the difficulties patients experience dealing with mental healthcare providers and also in expressing themselves when discussing intimate events in their lives with virtual strangers. There is no question that clinicians need to be more empathetic and that they ought to communicate more effectively with patience who come from a variety of different backgrounds and have different levels of understanding of their own psychology. For that reason alone, this paper is eminently publishable.

**Author response:** Many thanks for this very kind comment. We really appreciate it.

**Reviewer comment:** The authors also do an excellent job of describing the various reasons why a diagnosis might be missed but it would certainly be useful to understand how the diagnosis finally came to be made. Do the clinical records record the patient's level of understanding, the clinicians appreciation of the accuracy of the patients representations, and the difficulties that clinicians might have in generating in accurate clinical history?

**Author response:** Unfortunately we do not have access to this information. We had ethical approval to recruit via the registry and clinical referral but did not have approval to access medical notes beyond this. We have added a comment on this in the discussion.

**Reviewer comment:** Is it true that clinicians failed to query the presence of psychotic symptoms? Patients are ordinarily given symptom checklists or rating scales to respond to and most of those ask about psychotic symptoms. Not all of them do, however. Rating scales and symptom checklists, however, are good ways to make sure all of the bases are covered when one is taking a medical history and generating a proper diagnosis.

**Author response:** We agree that it is common for checklists and rating scales to be used. However, no respondent mentioned these. Additionally, if psychotic symptoms were highlighted on a checklist, it is likely that this would be followed-up with further discussion. It was common for participants to report that psychotic symptoms were not always asked about. But we have discussed the issue of memory and recall as a limitation in case this is the reason for this finding: “Further, service users' retrospective accounts are subject to distortions and recall bias, especially when the majority of service users had been diagnosed more than 3 years before the interview.”

**Reviewer comment:** The authors correctly identify memory problems that patients may have, especially when the clinical encounters may have occurred three years earlier. They might also emphasize the cognitive disorganization that frequently accompanies severe depression, especially major depression with psychosis. One doesn't wish to discount the accuracy of patients’ reports but human memory is inevitably fallible and it is possible, even in the context of semi structured interview, for the interviewers presuppositions to influence patients’ responses during a research interview.

**Author response:** Thanks for this very useful comment. We have discussed this in the first paragraph of the discussion (difficulty retelling the story) and have now expanded on this in the limitations: “In relation to this, cognitive issues and confusion (as mentioned above) at the time of presentation may have led to poor recall of events”.

**Reviewer comment:** My only important recommendation to the authors is to inform readers how the proper diagnosis of PMDD was ultimately made and to review patients’ commentaries in that light.

**Author response:** Unfortunately, as mentioned above, we do not have access to the medical records for the participants so are unable to do this. However, we have added this as a limitation of the study: “Similarly, lack of access to clinical notes means exploration of clinician’s difficulty in generating a diagnosis, or the patient’s understanding of their diagnosis as assessed by the clinical team was not possible.”

**Reviewer comment:** The topic is interesting, but Why u choice this topic

**Author response:** Many thanks. We believe this is sufficiently described in the introduction in where we describe the issues surrounding diagnosis in people with PMD.

**Reviewer comment:** In the introduction part, you should only focused about psychotic major depression

**Author response:** This is what has been done. Please clarify what parts you feel are not appropriate.

**Reviewer comment:** What tools do you used to assess psychotic major depression?

**Author response:** Diagnosis of PMD is confirmed in clinical interview using ICD or DSM criteria as described in the introduction. There is no validated tool for this.

**Reviewer comment:** What is the difference between major depression disorder with psychotic features, schizoaffective disorder and psychotic major depression?

**Author response:** Major depressive disorder with psychotic features is the same as Psychotic Major Depression. We have described this in the introduction: “Psychotic Major Depression (PMD) is defined in ICD-11 as depressive disorders with psychotic symptoms…”. The differences between PMD and schizoaffective disorder are not the focus of this work but have been described by others ([https://academic.oup.com/schizophreniabulletin/article-abstract/33/4/877/1930127?redirectedFrom=fulltext](https://eur03.safelinks.protection.outlook.com/?url=https%3A%2F%2Facademic.oup.com%2Fschizophreniabulletin%2Farticle-abstract%2F33%2F4%2F877%2F1930127%3FredirectedFrom%3Dfulltext&data=05%7C02%7Cmargaret.heslin%40kcl.ac.uk%7C9d71d7f85ce4498e6ae108dd30bb1383%7C8370cf1416f34c16b83c724071654356%7C0%7C0%7C638720302215317667%7CUnknown%7CTWFpbGZsb3d8eyJFbXB0eU1hcGkiOnRydWUsIlYiOiIwLjAuMDAwMCIsIlAiOiJXaW4zMiIsIkFOIjoiTWFpbCIsIldUIjoyfQ%3D%3D%7C0%7C%7C%7C&sdata=kZTit4v93wd1LFEDF1caEhgorP%2BHKvMmoKdKPlxgCbQ%3D&reserved=0))

**Reviewer comment:** Is schizoaffective disorder or not?

**Author response:** No it is not – please see ICD and DSM definition, and the above reference.

**Reviewer comment:** To Dx a patient with psychotic major depression is based- on DSM-5 or not, if not by what criteria will be diagnosis with psychotic major depression?

**Author response:** ICD-10 diagnoses were used – this has been added to the methods.

**Reviewer comment:** Is new finding on the management of major depression disorder with psychotic features, psychotic major depression and schizoaffective disorder? If yes what is new finding on the management of psychotic major depression?

**Author response:** This is not relevant to this paper.

**Reviewer comment:** In conclusions part: Psychotic major depression should be actively considered as a differential diagnosis by healthcare professionals when assessing an individual for unipolar depression, it is not understand, to me my 1st DDX will be schizoaffective disorder or bipolar 1 disorder with psychotic feature.

**Author response:** This conclusion follows on from the fact that participants reported that they approached services with depression and/or anxiety so the psychotic symptoms were sometimes overlooked. This is described in the discussion earlier: “Service users often approached services with “depression and/or anxiety”, and therefore, it is not surprising that health professionals did not elicit psychotic phenomena via interview.”

**Reviewer comment:** References, there problems on references examples Hales [11] suggests that because patients recognise that their thought patterns are unusual, they may attempt to conceal their symptoms from others…..

**Author response:** Please can you clarify the issue – reference 11 corresponds to Hales so we cant spot what the issue is.

**Reviewer comment:** Which tools were used to assess barriers, and stigma…

**Author response:** None. This is a qualitative piece of work based on semi-structured interviews as per the methods. No tools were used.

**Reviewer comment:** Write your Source of population,Study population, and  study unit

**Author response:** This has been described in detail in the participants and recruitment section.

**Reviewer comment:** What sampling methods and technique were used to asses

**Author response:** This has been described in detail in the recruitment section.

**Reviewer comment:** Why only 10 people participated in the interviews?

**Author response:** Smaller sample sizes are appropriate for qualitative work such as this (Patton MQ. Enhancing the quality and credibility of qualitative analysis. Health services research. 1999;34(5):1189). Additionally, we have discussed the sample size in the strengths and limitations section: “Although the sample size was small, long and extensive interviews were conducted, which provided a large amount of in-depth data.”

**Reviewer comment:** Service user frustration with diagnostic instability and lack of certainty, what does it mean

**Author response:** This is described in the results text section “Service user frustration with diagnostic instability and lack of certainty” in detail.

**Reviewer comment:** In the Discussion part, there is no references, so put references and discuss with different studies

**Author response:** This study is unique and has not done before so there is nothing to compare it with. We believe we have extensively discussed the findings.

**Reviewer comment:** How they know whether the diagnoses were psychotic major depression or not?

**Author response:** As described in the methods, all participants: “had a formal recorded clinical diagnosis of PMD”

**Reviewer comment:** What does it mean medical malpractice?

**Author response:** Apologies, we cannot find a reference to this in the paper – please can you clarify where this is?

**Reviewer comment:** Why you exclude individuals above 64 years old from the study?

**Author response:** Sometimes, psychotic disorders and dementia can be confused ([https://alz-journals.onlinelibrary.wiley.com/doi/10.1016/j.trci.2017.06.001](https://eur03.safelinks.protection.outlook.com/?url=https%3A%2F%2Falz-journals.onlinelibrary.wiley.com%2Fdoi%2F10.1016%2Fj.trci.2017.06.001&data=05%7C02%7Cmargaret.heslin%40kcl.ac.uk%7C9d71d7f85ce4498e6ae108dd30bb1383%7C8370cf1416f34c16b83c724071654356%7C0%7C0%7C638720302215342033%7CUnknown%7CTWFpbGZsb3d8eyJFbXB0eU1hcGkiOnRydWUsIlYiOiIwLjAuMDAwMCIsIlAiOiJXaW4zMiIsIkFOIjoiTWFpbCIsIldUIjoyfQ%3D%3D%7C0%7C%7C%7C&sdata=CIT2BLMlusdrUM5Gf1KhPKVqlRkArFtHkh2dkCSs3aQ%3D&reserved=0)). Therefore, we excluded individuals over the age of 64 to prevent misdiagnosis of dementia as psychotic major depression (PMD), which could potentially confound the results.

**Edits made for journal requirements:**

1. When submitting your revision, we need you to address these additional requirements.

Please ensure that your manuscript meets PLOS ONE's style requirements, including those for file naming. The PLOS ONE style templates can be found at 
[https://journals.plos.org/plosone/s/file?id=wjVg/PLOSOne_formatting_sample_main_body.pdf](https://eur03.safelinks.protection.outlook.com/?url=https%3A%2F%2Fjournals.plos.org%2Fplosone%2Fs%2Ffile%3Fid%3DwjVg%2FPLOSOne_formatting_sample_main_body.pdf&data=05%7C02%7Cmargaret.heslin%40kcl.ac.uk%7C9d71d7f85ce4498e6ae108dd30bb1383%7C8370cf1416f34c16b83c724071654356%7C0%7C0%7C638720302215362665%7CUnknown%7CTWFpbGZsb3d8eyJFbXB0eU1hcGkiOnRydWUsIlYiOiIwLjAuMDAwMCIsIlAiOiJXaW4zMiIsIkFOIjoiTWFpbCIsIldUIjoyfQ%3D%3D%7C0%7C%7C%7C&sdata=z2EWDLgrSaU09A2HaiMCIr6fnzYhvwMVRxdBO3wMcGs%3D&reserved=0) and 
[https://journals.plos.org/plosone/s/file?id=ba62/PLOSOne_formatting_sample_title_authors_affiliations.pdf](https://eur03.safelinks.protection.outlook.com/?url=https%3A%2F%2Fjournals.plos.org%2Fplosone%2Fs%2Ffile%3Fid%3Dba62%2FPLOSOne_formatting_sample_title_authors_affiliations.pdf&data=05%7C02%7Cmargaret.heslin%40kcl.ac.uk%7C9d71d7f85ce4498e6ae108dd30bb1383%7C8370cf1416f34c16b83c724071654356%7C0%7C0%7C638720302215388405%7CUnknown%7CTWFpbGZsb3d8eyJFbXB0eU1hcGkiOnRydWUsIlYiOiIwLjAuMDAwMCIsIlAiOiJXaW4zMiIsIkFOIjoiTWFpbCIsIldUIjoyfQ%3D%3D%7C0%7C%7C%7C&sdata=Lbh05wvD08c9kKzn3QB1IA%2FS18E%2FYeUUzgE83WUV7LI%3D&reserved=0)

**Author response:** Addressed.

2. Thank you for stating the following in the Acknowledgments Section of your manuscript: 
This paper represents independent research funded by the National Institute for Health and Care Research (NIHR) Biomedical Research Centre at South London and Maudsley NHS Foundation Trust in partnership with King’s College London. The views expressed are those of the author(s) and not necessarily those of the NHS, the NIHR or the Department of Health and Social Care. We acknowledge and are very grateful for the support of the Maudsley Biomedical Research Centre’s Young Person’s Mental Health Advisory Group, and the Maudsley Biomedical Research Centre’s Service User Advisory Group.
Professor Young’s independent research is funded by the National Institute for Health and Care Research (NIHR) Maudsley Biomedical Research Centre at South London and Maudsley NHS Foundation Trust and King's College London. The views expressed are those of the author(s) and not necessarily those of the NIHR or the Department of Health and Social Care.

We note that you have provided funding information that is not currently declared in your Funding Statement. However, funding information should not appear in the Acknowledgments section or other areas of your manuscript. We will only publish funding information present in the Funding Statement section of the online submission form. 
Please remove any funding-related text from the manuscript and let us know how you would like to update your Funding Statement. Currently, your Funding Statement reads as follows: 
The author(s) received no specific funding for this work.

Please include your amended statements within your cover letter; we will change the online submission form on your behalf.

**Author response:** This should be: “The author(s) received no specific funding for this work”

3. Thank you for stating the following in the Competing Interests section: 
MH reports funding from NIHR. AY reports funding from Flow Neuroscience, Novartis, Roche, Janssen, Takeda, Noema pharma, Compass, Astrazenaca, Boehringer Ingelheim, Eli Lilly, LivaNova, Lundbeck, Sunovion, Servier, Livanova, Janssen, Allegan, Bionomics, Sumitomo Dainippon Pharma, Sage, Neurocentrx, NIMH, CIHR, NARSAD, Stanley Medical Research Institute, MRC, Wellcome Trust, Royal College of Physicians, BMA, MSFHR, EU Horizon 2020, NIHR. No other authors reported competing interests.

We note that you received funding from a commercial source: 
Please provide an amended Competing Interests Statement that explicitly states this commercial funder, along with any other relevant declarations relating to employment, consultancy, patents, products in development, marketed products, etc.

Within this Competing Interests Statement, please confirm that this does not alter your adherence to all PLOS ONE policies on sharing data and materials by including the following statement: "This does not alter our adherence to PLOS ONE policies on sharing data and materials.” (as detailed online in our guide for authors [http://journals.plos.org/plosone/s/competing-interests](https://eur03.safelinks.protection.outlook.com/?url=http%3A%2F%2Fjournals.plos.org%2Fplosone%2Fs%2Fcompeting-interests&data=05%7C02%7Cmargaret.heslin%40kcl.ac.uk%7C9d71d7f85ce4498e6ae108dd30bb1383%7C8370cf1416f34c16b83c724071654356%7C0%7C0%7C638720302215413776%7CUnknown%7CTWFpbGZsb3d8eyJFbXB0eU1hcGkiOnRydWUsIlYiOiIwLjAuMDAwMCIsIlAiOiJXaW4zMiIsIkFOIjoiTWFpbCIsIldUIjoyfQ%3D%3D%7C0%7C%7C%7C&sdata=LHeaNFHAFMTIMx0sLx7X56LuWUX2yvj6l0FERbUq5u0%3D&reserved=0)).  If there are restrictions on sharing of data and/or materials, please state these. Please note that we cannot proceed with consideration of your article until this information has been declared.

Please include your amended Competing Interests Statement within your cover letter. We will change the online submission form on your behalf.

**Author response:** Competing Interests Statement: This does not alter our adherence to PLOS ONE policies on sharing data and materials.

4. When completing the data availability statement of the submission form, you indicated that you will make your data available on acceptance. We strongly recommend all authors decide on a data sharing plan before acceptance, as the process can be lengthy and hold up publication timelines. Please note that, though access restrictions are acceptable now, your entire data will need to be made freely accessible if your manuscript is accepted for publication. This policy applies to all data except where public deposition would breach compliance with the protocol approved by your research ethics board. If you are unable to adhere to our open data policy, please kindly revise your statement to explain your reasoning and we will seek the editor's input on an exemption. Please be assured that, once you have provided your new statement, the assessment of your exemption will not hold up the peer review process.

**Author response:** This has been updated.
